# Clinical and molecular study of radiation-induced gliomas

**DOI:** 10.1038/s41598-024-53434-0

**Published:** 2024-02-07

**Authors:** Katerina Trkova, David Sumerauer, Adela Bubenikova, Lenka Krskova, Ales Vicha, Miroslav Koblizek, Josef Zamecnik, Bruno Jurasek, Martin Kyncl, Bela Malinova, Barbora Ondrova, David T. W. Jones, Martin Sill, Martina Strnadova, Lucie Stolova, Adela Misove, Vladimir Benes, Michal Zapotocky

**Affiliations:** 1 Prague Brain Tumor Research Group, Second Faculty of Medicine, Charles University and University Hospital Motol, V Uvalu 84, 15006 Prague 5, Czech Republic; 2 Center for Pediatric Neuro-Oncology, University Hospital Motol, V Uvalu 84 , 15006 Prague 5, Czech Republic; 3 Department of Pediatric Hematology and Oncology, Second Faculty of Medicine, Charles University Prague and University Hospital Motol, V Uvalu 84, 15006 Prague 5, Czech Republic; 4 Department of Pathology and Molecular Medicine, Second Faculty of Medicine, Charles University Prague and University Hospital Motol, V Uvalu 84, 15006 Prague 5, Czech Republic; 5 Department of Radiology, Second Faculty of Medicine, Charles University Prague and University Hospital Motol, V Uvalu 84, 15006 Prague 5, Czech Republic; 6 Department of Neurosurgery, Second Faculty of Medicine, Charles University Prague and University Hospital Motol, V Uvalu 84, 15006 Prague 5, Czech Republic; 7 Department of Oncology, Second Faculty of Medicine, Charles University Prague and University Hospital Motol, V Uvalu 84, 15006 Prague 5, Czech Republic; 8 Proton Therapy Center Czech, Budínova 1a, 180 00 Prague, Czech Republic; 9 Division of Pediatric Glioma Research, Hopp Children’s Cancer Center (KiTZ), Heidelberg, Germany; 10 National Center for Tumor Diseases (NCT), NCT Heidelberg, a partnership between DKFZ and Heidelberg University Hospital, Heidelberg, Germany; 11 German Cancer Research Center (DKFZ), Heidelberg, Germany

**Keywords:** CNS cancer, Paediatric cancer, CNS cancer

## Abstract

In this study, we provide a comprehensive clinical and molecular biological characterization of radiation-induced gliomas (RIG), including a risk assessment for developing gliomas. A cohort of 12 patients who developed RIG 9.5 years (3–31 years) after previous cranial radiotherapy for brain tumors or T-cell acute lymphoblastic leukemia was established. The derived risk of RIG development based on our consecutive cohort of 371 irradiated patients was 1.6% at 10 years and 3.02% at 15 years. Patients with RIG glioma had a dismal prognosis with a median survival of 7.3 months. We described radiology features that might indicate the suspicion of RIG rather than the primary tumor recurrence. Typical molecular features identified by molecular biology examination included the absence of *Histon3* mutation, methylation profile of pedHGG-RTK1 and the presence of recurrent *PDGFRA* amplification and *CDKN2A/B* deletion. Of the two long-term surviving patients, one had gliomatosis cerebri, and the other had pleomorphic xanthoastrocytoma with BRAF V600E mutation. In summary, our experience highlights the need for tissue diagnostics to allow detailed molecular biological characterization of the tumor, differentiation of the secondary tumor from the recurrence of the primary disease and potentially finding a therapeutic target.

## Introduction

Radiotherapy (RT) is an essential component of therapy for both solid and, mainly in the past, hematological malignancies in the pediatric population. RT improves the outcome of pediatric patients but is also associated with long-term risks. These can be observed especially in children with long-term follow-up^1^. One of the most serious risks is the development of radiation-induced gliomas (RIGs), which have been described in patients primarily treated mainly for acute lymphoblastic leukemia (ALL) and central nervous system (CNS) malignancies by cranial RT^2,3^. Anamnestic information about previous radiotherapy of the cranium in the treatment of the primary diagnosis is always crucial in the management of subsequent diagnostic procedures. It is necessary to use advanced diagnostic tools to reliably distinguish them from the recurrence of primary CNS tumors or from primary high-grade glioma. Radiological features to distinguish RIG from sporadic tumors have not yet been comprehensively described. Consequently, most RIG histologically fulfill the characteristics of high-grade gliomas, and it is difficult to distinguish them from their primary counterparts at this diagnostic level^4,5^. However, significant progress is now being made in their molecular biological characterization^6,7^. Unlike their primary counterparts, RIG do not usually carry the typical mutations in the *Histon3, IDH1/2 or BRAF* genes^1,8,9^*.* Based on methylation profiling, RIG mostly clustered within the GBM_pedRTK1 methylation group. In addition to *TP53* mutations, *PDGFRA* amplification and *CDKN2A/B* deletion are the most frequently described molecular alterations in RIG^6,7^.

RIGs are characterized by an aggressive clinical course with a poor prognosis^1,3,10^. Treatment regimens are not defined and include modalities used to treat primary high-grade gliomas. Unfortunately, these procedures have no curative potential in the majority of patients^10^. Here, we performed a single-institution retrospective study of 12 RIG patients with complete clinical, imaging and comprehensive molecular-biological data. We have attempted to define the radiological characteristics of RIG and to present detailed clinical information on each case, including two rare cases of RIG long-term survivors. We present comprehensive genetic and epigenetic data of the examined tumor samples. Due to the availability of follow-up in patients from the primary treatment cohort, we present a unique statistical dataset on the risk of developing RIG in irradiated patients.

## Materials and methods

### Patient cohort and tumor samples

This study was conducted upon ethics approval (Institutional Ethics Committee of the Second Faculty of Medicine Charles University in Prague 17.6.2020). The authors of this publication declare that they have obtained the informed consent of the patient's legal representatives for the publication of their anonymized data for this study. All methods were performed in accordance with the relevant guidelines and regulations.

Patients with RIG located in the radiation field and fulfilling the Cahan’s criteria were identified^11^. Patients’ charts were reviewed to obtain demographic information, primary tumor histology, tumor location and histology at the time of RIG diagnosis. Archival tissue blocks of the RIG tumors were retrieved and used for subsequent molecular analyses as detailed below.

### DNA extraction and direct sequencing

The most representative tissue blocks, containing the maximum percentage of tumor tissue, were selected by the pathologist. Genomic DNA was extracted from each formalin-fixed, paraffin-embedded (FFPE) tissue block using a QIAamp DNA FFPE Tissue Kit (Qiagen, Germany) or from fresh frozen sections using TRIzol Reagent (Life Technologies, Merelbeke, Belgium). The hotspot mutations at codons 27 and 34 of *H3F3A*, codon 27 of *HIST1H3B*, codon 600 of *BRAFex15*, codons 546 and 656 of *FGFR1ex12*, and codon of *FGFRex14* were examined using previously described primer pairs^12–14^. Amplification was performed using 2 × PCRBIO HS Taq Mix Red (PCR Biosystems Ltd., London, UK). The PCR products were electrophoresed in a 1.5% agarose gel and recovered using the Gel DNA Fragments Extraction Kit (Geneaid, Taiwan). Direct Sanger sequencing was performed using BigDye Terminator v 3.1 chemistry (Life Technologies) and an ABI PRISM 3130 genetic analyzer (Applied Biosystems). The results were analyzed using Chromas lite 2.01 (Technelysium, Pty Ltd., Brisbane, Australia).

### Genome-wide DNA methylation profiling

DNA methylation was evaluated in eight RIG with the Infinium MethylationEPIC BeadChip Kit (Illumina, San Diego, CA, USA). A total of 250 ng of DNA from fresh frozen tumor tissue or FFPE was treated with bisulfite conversion using the ZymoResearch EZ DNA Methylation kit (Zymo Research Corp, Irvine, CA, USA). The Infinium HD Methylation Assay was performed according to the manufacturer’s explicit specifications. The methylation class was established using web-based analysis via https://www.molecularneuropathology.org/. Copy number variation (CNV) analysis was performed by the conumee Bioconductor package^15^.

T-SNE analysis was performed as described previously using a reference cohort of primary HGG subgroups as well as a published RIG dataset by Deng et al.^7,15^.

### Next-generation sequencing

The DNA NGS VariantPlex HS Solid Tumor kit (Archer) (Supplementary data) was used following the manufacturer´s instructions. DNA was extracted from FFPE sections (QIAamp DNA FFPE Tissue kit, Qiagen) followed by library preparation. Anchored Multiplex polymerase chain reaction amplicons were sequenced on an Illumina MiSeq, and the data were analyzed using Archer software.

### Radiology

All patients were scanned either during scheduled follow-up sessions or extraordinarily in cases of neurological symptoms. Various MRI scanners certified for diagnostic imaging have been used, all of which use a 1.5 Tesla magnetic field. In all cases, the examination protocol included T1- and T2-weighted imaging, FLAIR imaging, DWI/ADC evaluation and T1-weighted imaging with gadolinium-based contrast agent (GBCA) administration. MRI findings were evaluated within the central tumor board by a radiology expert.

### Statistics

The normality of the data was evaluated according to Shapiro‒Wilk’s test. Overall survival analysis was analyzed according to the Kaplan‒Meier method with p values derived from the log-rank test. The hazard model function in time-to-event analysis was applied for the evaluation of mortality and RIG-development risk over time. All calculations were performed in the open-source R environment (v4.1.2). Graphical interpretations were modeled in OriginPro software (OriginLab Corporation).

## Results

### Demographics and cumulative risk of RIG development

Between 2000 and 2022, a total of 12 RIG patients were diagnosed at our institution. All patients had received previous treatment with RT for a primary CNS tumor or T-ALL. Three of these patients were originally treated in the 1980s and 1990s. One patient was referred from abroad. Remaining 8 patients were diagnosed and treated for primary malignancy between 2000 and 2015. All 12 patients were used for survival analysis, radiological analysis and molecular profiling. The RIG patient group consisted of six males and six females. Their primary diagnoses comprised T-ALL (*n* = 3), ependymoma (*n* = 4), choroid-plexus carcinoma (*n* = 1), medulloblastoma (*n* = 2), meningioma (*n* = 1) and skull chondrosarcoma (*n* = 1). Age at the primary diagnosis ranged between 2 and 11 years (median 9 years). Patients underwent photon radiotherapy at doses ranging from 12 to 59.4 Gy (median 50.65 Gy). They developed RIG in the median 9.5 years after the primary diagnosis in the range of 3 to 31 years. All RIGs were located in the radiation field and fullfiled the Cahan’s criteria^11^. RIG tumor samples were histologically reported as glioblastoma gr. 4 (*n* = 7), anaplastic astrocytoma gr. 3 (*n* = 1), anaplastic ganglioglioma gr. 3 (*n* = 1), gliomatosis cerebri gr. 3 (*n* = 1) and embryonal high-grade tumors (*n* = 2). The clinical, demographic and molecular biological characteristics of the RIG patients are summarized in Table 1.

**Table 1:** Clinical, demographic and molecular data.

ID	Primary diagnosis	RT dose (Gy) (Fractions)	Age primary Dx (y)	Age RIG (y)	RIG OS (m)	RIG location	Histology	Methylation class	CS	CNV	SNV	Therapy
RIG1	EPE	50.4* (28 × 1.8)	11	29	5	PF	GBM	RTK1c	0.9	neg	ND	50.4 Gy (28 × 1.8), TMZ
RIG2	CPC	54 (30 × 1.8)	6	11	3	Right thalamus	GBM	RTK1c	0.28	CDKN2A del, MYCN amp	ND	TMZ
RIG3	EPE	54* (30 × 1.8)	9	18	3	PF	AA	no match	< 0.3	ND	No SNV	COMBAT
RIG4	MBL	59.4* (33 × 1.8)	10	21	6	PF	HG ET	RTK1c	0.67	PDGFRA amp, CDKN2A del	No SNV	MEMMAT
RIG5	T-ALL	18 (12 × 1.5)	7	11	8	Right F lobe	GBM	RTK1b	1	neg	No SNV	None
RIG6	T-ALL	12 (8 × 1.5)	10	20	alive (108)	Both hemispheres	GC	failed	ND	ND	ND	50.4 Gy (28 × 1.8), TMZ
RIG7	MBL	55.8* (31 × 1.8)	11	15	10	PF	GBM	RTK1c	0.45	PDGFRA amp, CDK4 amp	ROS1 R2035C	45 Gy (25 × 1.8), TMZ
RIG8	EPE	59.4 (33 × 1.8)	2	7	12	PF	GBM	RTK1c	0.96	CDKN2A del	PIK3CA D454_P458del insA	BVZ
RIG9	T-ALL	12 (8 × 1.5)	5	9	15	Left P lobe	HG ET	RTK1c	0.99	PDGFRA amp	No SNV	54 Gy (30 × 1.8), TMZ
RIG10	MNG	45 (SRS)	9	23	alive (99)	Left T lobe	aGG	PXA	0.99	CDKN2A del	BRAF V600E	RT/CHT
RIG11	EPE	50.4 (28 × 1.8)	5	37	3	Right P lobe	GBM	ND	ND	ND	ND	60 Gy (30 × 2), TMZ
RIG12	CSa	50.4 (28 × 1.8)	10	45	2	Right T lobe	GBM	RTK1	0.73	PDGFRA amp, CDKN2A del	TP53 c.560-1G >APTEN R130*	None

Radiation doses marked with * indicate that craniospinal radiation was used.

AA—anaplastic astrocytoma grade 3, aGG—anaplastic ganglioglioma grade 3, CNV—copy number variant, CPC—choroid plexus carcinoma, COMBAT—combined metronomic low dose biodifferentiating antiangiogenic therapy, CSa—chondrosarcoma, Dx—diagnosis, EPE—ependymoma, F frontal, GC—gliomatosis cerebri, GBM—glioblastoma, Gy—gray, HG ET—high grade embryonal tumor, m—month, MBL—medulloblastoma, MEMMAT—Medulloblastoma European Multitarget Metronomic Anti-Angiogenic Trial, MNG—meningioma, NA—not available, ND—not done, P—parietal, PF—posterior fossa, PXA—Pleomorphic xanthoastrocytoma, RIG—radiation-induced glioma, RTK1c—Glioblastoma, pediatric RTK1 type, subtype C, RTK1b—Glioblastoma, pediatric RTK1 type, subtype B, SNV—single nucleotide variant, SRS—stereotactic radiosurgery, T—temporal, T-ALL—T-cell acute lymphoblastic leukemia, TMZ—temozolomide, Y—year.

**Figure 1:**
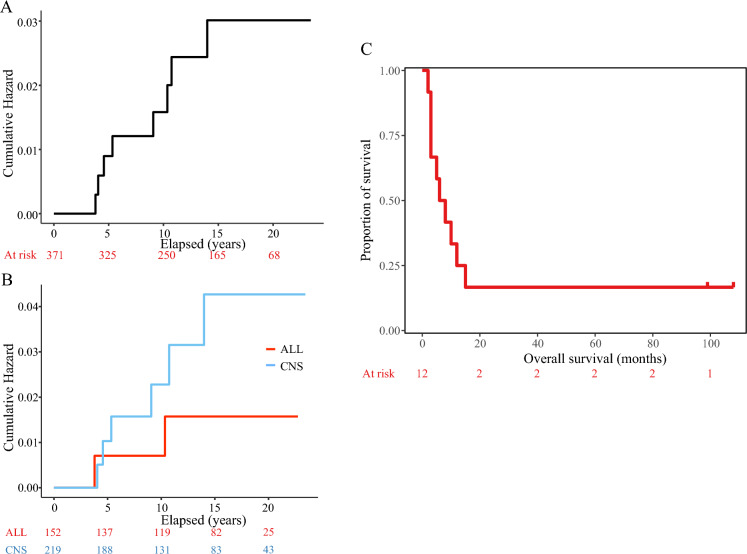
Risk analysis of the development of RIG and overall survival analysis. (**A**) Overall hazard function evaluating the risk of RIG development within the primary cohort after RT. (**B**) Analogous demonstration of the risk of RIG development when divided into two groups based on primary diagnosis (CNS group (*n* = 219) vs ALL group (*n* = 152)). (**C**) Overall survival of patients with RIG.

To calculate the cumulative risk of RIG development after RT, a single institutional cohort of patients with irradiated craniums between 2000 and 2015 was established consisting of 371 cases (219 primary brain tumors and 152 acute lymphoblastic leukemias) with follow-up censored by the end of 2022. Median time of follow-up was 13.3 years. Eight out of our 12 patients were treated within this range and developed RIG (six after brain tumor and two after ALL). Based on hazard functions, the derived risk of RIG development was 1.60% at 10 years and 3.02% at 15 years (Fig. 1A). The hazard difference between the CNS tumor group and leukemias was not significantly different (*p* = 0.72) (Fig. 1B). Table 2 displays the increasing risk of developing RIG with the time elapsed since the primary diagnosis.

**Table 2:** Derived risks of RIG development based on Hazard Function seen in Fig. 1A.

Years	Risk of RIG development (%)
3	0.28
4	0.89
5	1.20
9	1.60
11	2.45
13	3.02

Furthermore, we aimed to estimate the prevalence of RIG among late events occurring later than 3 years after cranial radiation therapy. We identified all patients treated with radiation therapy for primary brain tumors between 2000 and 2015 (219 primary brain tumors as mentioned above). All intraaxial intracranial tumor recurrences/progressions and secondary malignancies occurring later than 3 years from radiation therapy were evaluated. Altogether, 30 patients with late events were identified, 28 of whom underwent histopathological verification of the subsequent tumor. The RIG diagnosis was confirmed in 6 patients, representing 21.4% of all histologically verified late events.

### Treatment and outcome of RIG

RIG therapy is very challenging because it affects a vulnerable population of patients previously treated for malignancy and dealing with various late effects and chronic medical conditions. The available treatment options are thus limited and sometimes cannot be used at all.

Similar to primary pediatric high-grade glioma, surgery is an essential component of treatment. Complete resection of the tumor was attempted and achieved in three cases (RIG1, 9, 10). In all other cases except for RIG8, only partial resection or biopsy was performed. In addition to surgery, patients were treated mostly by another course of radiotherapy combined with temozolomide chemotherapy (*n* = 6). RIG4, originally diagnosed by histology as an embryonal high-grade tumor, was treated by oral metronomic combined chemotherapy as per Medulloblastoma European Multitarget Metronomic Anti-Angiogenic Trial (MEMMAT)^16^. Patients RIG2 and RIG3, who were unfit to receive radiotherapy, were treated with a palliative oral chemotherapy regimen. In the case of RIG5 and RIG12, no anticancer treatment was initiated because of the very poor clinical and neurological condition of the patients. RIG8 was initially diagnosed due to the new occurrence of cranial nerve palsies as brainstem radiation necrosis 5 years after posterior fossa ependymoma irradiation. The diagnosis was based solely on MRI radiological appearance of brain stem involvement, and the patient was treated with repeated courses of bevacizumab. At the time of clinical deterioration and tumor confirmation on imaging, no further treatment was pursued, and the true histological origin of the tumor was discovered only postmortem.

There were two long-term survivors in our RIG cohort, patients RIG6 and RIG10. Patient RIG6 was diagnosed with T-ALL at the age of 10 years. She was exposed to cranial radiotherapy at a dose of 12 Gy at that time as per the protocol treatment^17^. She developed RIG, radiologically fitting to the diagnosis of gliomatosis cerebri, histopathologically described as anaplastic astrocytoma gr. 3 (IDH1 wild-type) from the needle biopsy. She then underwent radiotherapy to the affected brain areas to a dose of 50.4 Gy and concomitant chemotherapy with temozolomide. Unfortunately, only four months after the end of the radiotherapy course was progression of the disease into the frontal lobe detected on MRI. The patient was indicated for palliative reirradiation at a dose of 30.6 Gy and metronomic oral chemotherapy using the combined metronomic low dose biodifferentiating antiangiogenic therapy (COMBAT) regimen for 21 months^18^. Since then, the patient has had a stable disease (20 years from diagnosis of ALL and 7 years from the RIG diagnosis) in a remarkably good clinical condition.

RIG10 presented with a primary diagnosis of meningioma in the left temporal area. The tumor was resected, and a small residual tumor was irradiated by stereotactic radiosurgery (Leksell gamma knife) to a dose of 45 Gy. He developed RIG after 14 years, histopathologically described as anaplastic ganglioglioma WHO gr. 3 with BRAF V600E mutation. The tumor was resected, and the patient was treated with radiotherapy and concomitant chemotherapy with temozolomide. Seven years after the diagnosis of RIG, the patient´s tumor relapsed. Complete resection of the tumor recurrence was performed, and the patient was treated with temozolomide chemotherapy. No biological therapy with BRAF/MEK inhibitors has been attempted by the adult neuro-oncology service to date, but he has now been in second complete remission for more than 10 months.

Despite the two long-term survivors, the prognosis of the patients within the RIG cohort is rather dismal. In our cohort, ten patients died with a median survival of only 7.3 months after the diagnosis of RIG (Fig. 1C).

### Radiological characteristics

**Figure 2:**
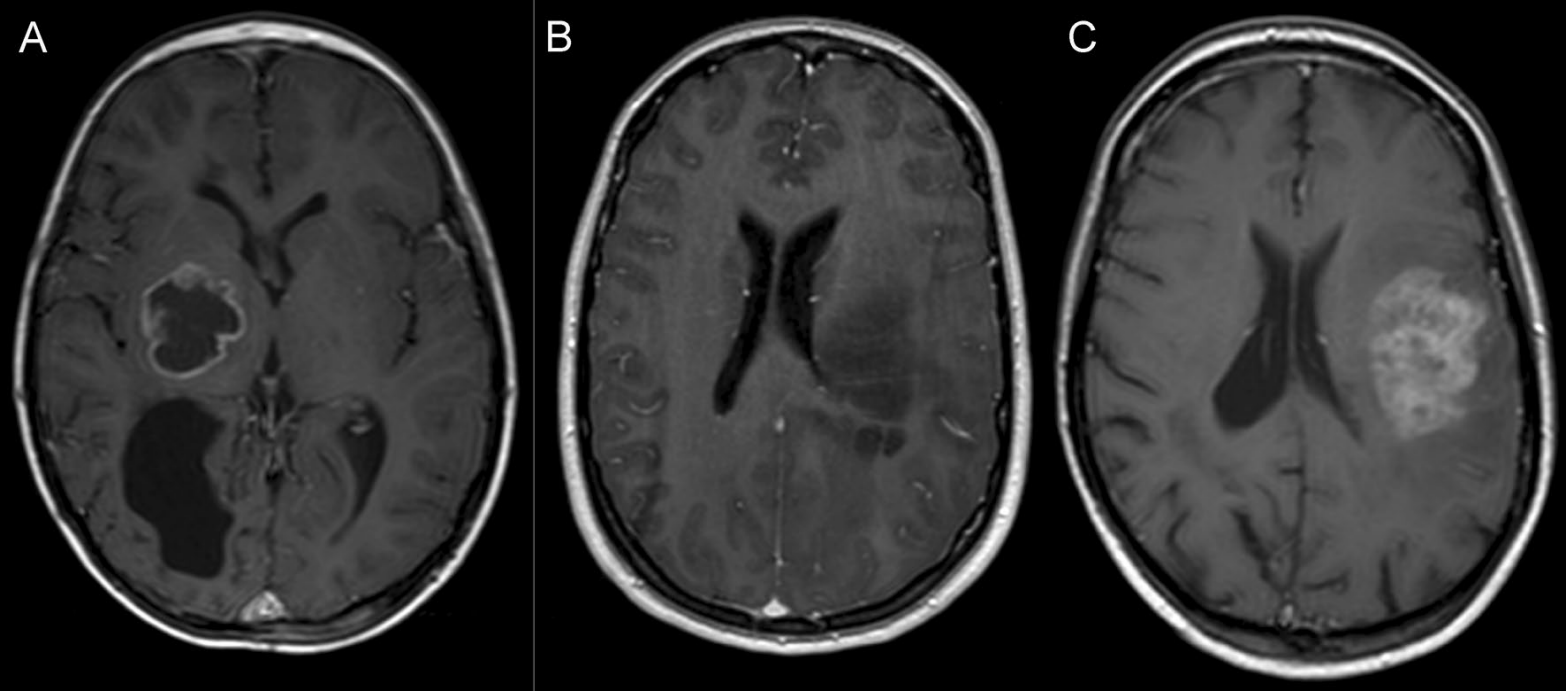
Radiological characteristics of RIG. Examples of radiological subgroups of RIG according to the character of contrast enhancement: (**A**) arcuate pattern enhancement—RIG2, (**B**) no enhancement—RIG6, (**C**) diffuse enhancement—RIG11.

The standard MRI sequences were reviewed to describe RIG imaging characteristics. We uncovered various patterns occurring in RIG patients. The first largest group (*n* = 7, RIG1, 2, 4, 5, 7, 9, 10, and 12) was represented by T1 hypo-intense and T2 hyperintense lesions with perilesional edema in FLAIR and peripheral contrast enhancement with an arcuate pattern. Furthermore, two other patients exhibited different contrast enhancement with remarkably diffuse patterns (RIG3 and 11). Finally, the third radiologically distinct tumors showed little or no contrast enhancement at the time of lesion detection (*n* = 2, RIG6 and 8) (Fig. 2).

### Molecular biology

**Figure 3:**
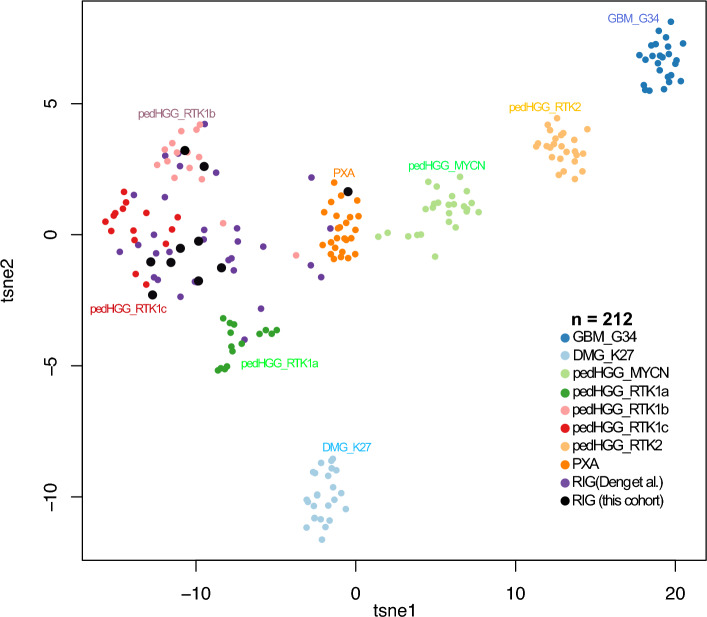
Molecular characterization of RIGs based on methylation profiling. T-SNE analysis demonstrated that the majority of RIG samples from our cohort clustered with the pedHGG-RTK1 subgroup. The previously published cohort of 32 RIG samples (Deng et al.) is shown here, which further validates our data. RIG samples were compared with a cohort of 170 reference samples of histologically and molecularly described CNS tumors. Abbreviations: PXA—pleomorphic xanthoastrocytoma; GBM_G34—Glioblastoma, H3.3 G34 mutant; DMG_K27—diffuse midline glioma H3 K27 mutant; pedHGG_MYCN—pediatric high-grade glioma subclass MYCN; pedHGG_RTK1a—Pediatric high-grade glioma, pediatric RTK1 type, subtype A; pedHGG_RTK1b—Pediatric high-grade glioma, pediatric RTK1 type, subtype B; pedHGG_RTK1c—Pediatric high-grade glioma, pediatric RTK1 type, subtype C; pedHGG_RTK2—Pediatric high-grade glioma, pediatric RTK2 type.

Out of 12 patients, 10 had a tissue sample available and sufficient quality DNA for the methylation array, 10 patients for direct sequencing, and 8 for next generation sequencing (NGS). Whole-genome DNA methylation profiling was performed to evaluate epigenetic differences between RIGs and primary pediatric high-grade gliomas. Heidelberg classifier v12.3 classified 8 samples as “Pediatric high-grade glioma, subclass RTK1” (pedHGG-RTK1) with calibrated scores (CS) over 0.9 in 5 cases and one sample as “Pleomorphic xanthoastrocytoma” with CS = 0.99. Two samples did not achieve a sufficient score to match any class. Interestingly, all pedHGG-RTK1 samples clustered with the methylation class “Glioblastoma IDH-wildtype, subclass midline” in the 11b4 version of the classifier with variable CS ranging from 0.21 to 0.99. Furthermore, t-SNE analysis was performed using a reference cohort and a previously published RIG cohort^7^. This demonstrated that our samples clustered with pedHGG-RTK1c (*n* = 7), pedHGG-RTK1b (*n* = 2), and PXA (*n* = 1) (Fig. 3).

Copy number variations (CNVs) inferred from DNA methylation data were analyzed to evaluate possible recurrent CNV changes among RIG. *PDGFRA* amplification was found in (*n* = 4, 44% of samples), *CDKN2A/B* homozygous deletion in (*n* = 5, 55.5% of samples). Other high-level amplifications were detected in single patients, such as *CDK4* amplification (RIG7) and *MYCN* amplification (RIG2). Recurrent chromosomal alterations were also observed, in particular 1p loss (*n* = 6; 66.6%), 1q gain (*n* = 5; 55.5%), and various partial 6q deletions (*n* = 4; 44.0%).

Proportion of our RIG cases presented with infiltration of midline structures; therefore, we performed direct sequencing focusing on mutations in *Hist1H3B* and *H3F3A* in all patients with available material (except for RIG6 and 11). Furthermore, none of these cases were positive for H3 K27M or H3 G34R mutations, which are usually present in a subset of pediatric HGG. Further sequencing involved other drivers typical for HGG, such as the *FGFR1*, *IDH1*, and *BRAF* genes. All samples tested wild-type except one sample positive for BRAF V600E corresponding with a case classified as PXA using a methylation array (RIG10).

In addition to the direct sequencing, DNA panel NGS was performed on samples with sufficient quality material (*n* = 8) and revealed additional somatic variants in three cases involving the *PIK3CA*, *PTEN* and *ROS1* genes (Table 1). Interestingly, somatic pathogenic variant in the *TP53* gene was detected in only one case (RIG12). Furthermore, no IDH1/IDH2 mutations were identified.

## Discussion

RIG represents serious late sequelae occurring 3–45 years after previous radiation therapy. Our data indicate that RIG represents a frequent late event after cranial irradiation. Strikingly, RIG represented over 20% of all intracranial intraaxial late events occurring later than 3 years after the diagnosis. Therefore, RIG should be excluded in all cases with suspected recurrence or progression after this time point. Furthermore, we presented a model estimating the risk of RIG development using a homogenous consecutive cohort treated in our center over a 15-year period. We have been able to estimate that the cumulative RIG risk will reach 3% after 15 years. Because our cohort and other published cohorts reported patients developing RIG even 35 to 45 years^19^ after radiation therapy, it is very likely that the actual risk of developing RIG exceeds 3% more than 15 years after the primary tumor RT. Consistent with our findings, the increasing cumulative incidence of RIG development was also demonstrated using SEER data^20^. Based on SEER data, the risk of RIG development was estimated to be between 1 and 4%, and RIG was responsible for 2 to 10% of all pediatric brain tumor deaths^20^.

The radiation doses at the primary diagnosis ranged from 12 to 59.4 Gy, demonstrating that even children who received as little as 12 Gy of neurocranium irradiation for leukemia were at risk of RIG development. This is in keeping with data from CCSS that demonstrated risk of secondary glioma in cases that received over 10 Gy brain irradiation. In the CCSS study, the peak odds ratio was identified at a radiation dose level of 30–44.9 Gy^21^.

RIG represented a disease with a dismal prognosis with a median overall survival of 7.3 months in our cohort. These results are similarly unfavorable as in primary H3-mutant pediatric HGG. In contrast to these data, two long-term survivors of RIG were reported. One of them was a patient with IDH1 wild-type gliomatosis cerebri that could not be further characterized. Despite the clear documented progression, in the long term (7 years after diagnosis), the disease was therapeutically stabilized by reirradiation (third cycle of radiotherapy for this patient). The second patient with a biologically more favorable tumor profile (PXA) was characterized by the BRAF V600E mutation. Small proportion of RIG tumors were reported to cluster with PXA. They harbor MAPK pathway alterations including genes *RAF1, NTRK2*, but also *BRAF*. Hotspot mutation BRAF V600E has never been reported in previous studies (Table 3).

**Table 3:** Molecular biology characteristic of RIG in the recent studies.

Study	Type of study	Patient number	Methylation class	Copy number variations	Focal somatic alterations	Gene fusions
DeSisto et al. (2021)	Multicentric	32	PedRTK1 (25/31) PXA (1/31)	1p loss (10/25), 1q gain (13/25), 13q loss (10/25), 14q loss (10/25),*PDGFRA* gain/amplification (11/31), *CDK4* amplification (6/31), *CDKN2A* loss (9/31), and *BCOR* loss (7/31)	*PDGFRA, CDKN2A, BCOR, BRAF, NF1, TP53, CDK4*	*MET* fusions
Deng et al. (2021)	Multicentric	32	PedRTK1 (29/32), PXA (3/32)	*PDGFRA* amplification (6/9 ALL-RIG; 11/23 MB- RIG), loss of *CDKN2A/B* (4/9 ALL-RIG; 17/23 MB-RIG)	*TP53, CBL, PDGFRA, NTRK2, EGFR, RAF1, ATRX, BCOR*	*PTPRZ1::MET, CAPZA2::MET, FYCO1::RAF1, GFAP1::NTRK2*
Whitehouse et. al (2021)	Metaanalysis	102	Not analyzed	*PDGFRA* amplification(10/21), *CDK4* amplification(4/10), *CDKN2A* deletion(13/28), 1q gain(53%), 1p loss(47%), 13q loss(59%)	*PDGFRA, TP53, ATRX, PTEN, PIK3CA, BRAF, IDH1*	*GTF2I::BRAF*
Trkova et al. (this study)	Single-centre	12	PedRTK1 (9/10), PXA (1/10)	*PDGFRA* amplification (4/9), *CDKN2A/B* deletion(5/9), *CDK4* amplification(1/9), *MYCN* amplification (1/9), 1p loss (6/9), 1q gain(1/9), 6q deletions (4/9)	*BRAF, ROS1, PIK3CA, TP53, PTEN*	not performed

Comprehensive radiological characterization of RIG is currently lacking in the literature. The uniqueness of our cohort lies in the fact that all patients were examined in one department by one radiology team. Therefore, for the first time, we can provide a comprehensive analysis of a cohort of patients with RIG.

On this basis, we want to emphasize the most common MRI pattern found in patients with radiotherapy-induced gliomas that should be taken into account when evaluating the brain MRI of patients with a history of cranial radiotherapy. If the lesion is peripherally contrast-enhancing with an arcuate pattern, while the lesion is hypointense in T1, hyperintense in T2 and shows perilesional edema in FLAIR, RIG should be part of the differential diagnosis. Nevertheless, other patterns of enhancement were observed in our cohort, suggesting that lack of arcuate enhancement does not exclude RIG diagnosis. Furthermore, it may be challenging to differentiate RIG from radiation necrosis, as they might present with similar MRI features.

Comprehensive molecular characterization of the tissue from RIG patients proved to be critical to establish correct diagnosis and to identify possible targets for novel therapies. Morphological diagnosis proved to be challenging, as demonstrated in our cohort, with several tumors being reported as a recurrence of the original diagnosis. Whole genome DNA methylation array and subsequent Heidelberg methylation classifier refined the diagnosis in those patients. Overall, RIG cases were classified as pedHGG-RTK1, PXA or no match with insufficient score. Therefore, some cases might benefit from further analysis (for example, t-SNE clustering) to confirm the correct methylation class. In our study, t-SNE analysis was able to cluster tumors reliably with pedHGG-RTK1 or PXA subgroups, including cases with very low calibrated scores. Combining our cohort with a previously published dataset demonstrated that all samples clustered with pedHGG-RTK1b, pedHGG-RTK1c or PXA. CNV analysis inferred from DNA methylation data demonstrated consistent findings with previous studies documenting a high prevalence of *PDGFRA* amplification and *CDKN2A* homozygous deletion^6,7,22^. In addition to these recurrent CNVs, some cases harbored complex CNV changes with amplifications of *CDK4* or *MYCN* genes. DNA sequencing revealed targetable somatic single nucleotide variants (SNVs) in four cases, including BRAF V600E (PXA methylation class) and pathogenic somatic variants in the *PIK3CA, PTEN* and *ROS1* genes. Deng et al. reported high priority targets obtained from RNA sequencing (RNAseq) of RIG samples, including *MET, RAF1* or *NTRK2* gene fusions. Unfortunately, the yields of RNA from our archival tissue were not sufficient to perform RNAseq. Nevertheless, our study and previously published data strongly indicate that comprehensive molecular evaluation of RIG tissue is of utmost importance. The combination of CNV analysis (for PDGFRA amplification), DNA sequencing (targetable driver SNVs) and RNAseq (targetable fusions) significantly increases the chance of uncovering high priority targets in this disease with a dismal prognosis (Table 3).

Although the risk model was provided, radiological and molecular features were evaluated, there are certain limitations to this study. This study is retrospective with relatively small number of patients because of the rarity of RIG. Much larger cohort would be required in order to stratify the risk of RIG development depending on the radiation dose, to determine the role of extent of resection or to further evaluate specific MRI features of this rare disease.

### Supplementary Information

Supplementary Information.

## Data Availability

The datasets generated and analyzed during the current study are available in the Mendeley Data repository, 10.17632/vpgtz9pzw8.1.
