# Supplementary material for: Clinical and molecular study of radiation-induced gliomas
Source: Sci Rep. 2024 Feb 7;14:3118. doi: 10.1038/s41598-024-53434-0 (PMC10850080; doi:10.1038/s41598-024-53434-0)
Supplement: Supplementary file 1 — Supplementary Information. [file 41598_2024_53434_MOESM1_ESM.pdf]

Supplementary data:

VariantPlex HS Solid Tumor panel

ABL1, AKT1, ALK, APC, ATM, AURKA, BRAF, CCNE1, CDH1, CDK4, CDKN2A, CSF1R, CTNNB1, DDR2, EGFR, ERBB2, ERBB3, ERBB4, ESR1, EZH2, FBXW7, FGFR1, FGFR2, FGFR3, FLT3, FOXL2, GNA11, GNAQ, GNAS, H3F3A, HNF1A, HRAS, IDH1, IDH2, JAK2, JAK3, KDR, KIT, KRAS, MAP2K1, MET, MLH1, MPL, NOTCH1, NPM1, NRAS, PDGFRA, PIK3CA, PIK3R1, PTEN, PTPN11, RB1, RET, RHOA, ROS1, SMAD4, SMARCB1, SMO, SRC, STK11, TERT, TP53, VHL
